# Deep Learning Approaches for Classification of Honeycombing and Ground Glass Opacity in Lung CT Imaging: A Systematic Review

**DOI:** 10.3390/bioengineering13101134

**Published:** 2026-09-28

**Authors:** Hairul Aysa Abdul Halim Sithiq, Liyana Shuib, Muneer Ahmad, Chermaine Deepa Antony, Mohsin Iftikhar, Peer Azmat Shah

**Affiliations:** 1 Department of Information Systems, Faculty of Computer Science and Information Technology, University of Malaya, Kuala Lumpur 50603, Malaysia; aysa@siswa.um.edu.my; 2 Department of Computing, University of Roehampton, Roehampton Lane, London SW15 5PH, UK; muneer.ahmad@roehampton.ac.uk; 3 Department of Biomedical Imaging, University Malaya Medical Centre, Kuala Lumpur 50603, Malaysia; chermaine@ummc.edu.my; 4 Faculty of Computer Information Science, Higher Colleges of Technology, Al-Faseel Campus, Fujairah P.O. Box 1626, United Arab Emirates; pshah@hct.ac.ae

**Keywords:** honeycombing lungs, ground-glass opacity, deep learning, convolutional neural networks, medical image classification, CT imaging, Resnet50v2, lung disease detection

## Abstract

Lung diseases such as Honeycombing (HC) and Ground Glass Opacity (GGO) present significant challenges in medical image analysis due to their overlapping visual characteristics and complex structural patterns in Computed Tomography (CT) images. These conditions often exhibit similar appearances to normal lung tissue, making accurate differentiation difficult and highly dependent on imaging quality and expert interpretation. This paper presents a systematic literature review of studies published between 2011 and 2026 on lung disease detection and classification using medical imaging. A total of 98 selected studies is analyzed to examine recent developments in Machine Learning (ML) and Deep Learning (DL) techniques for automated lung disease analysis. The review covers key aspects including imaging modalities such as CT and chest X-ray, publicly available and private datasets, preprocessing techniques, feature extraction methods, classification models, and evaluation metrics. The findings indicate that traditional machine learning approaches were widely used in earlier studies, while recent research has increasingly shifted towards deep learning methods due to their ability to automatically learn discriminative features from medical images. Among these approaches, Convolutional Neural Networks (CNNs), ResNet-based architectures, DenseNet, and EfficientNet are the most commonly adopted models for lung disease classification. Despite significant advancements, several challenges remain, including limited availability of publicly accessible datasets, class imbalance, variability in imaging protocols, and lack of standardized evaluation frameworks. In addition, misclassification between visually similar conditions such as Honeycombing and GGO remains a persistent issue that affects diagnostic reliability. The review identifies key research gaps and highlights the need for standardized datasets, improved preprocessing techniques, and consistent evaluation protocols. Future research should focus on enhancing model generalisation and robustness across diverse datasets to support reliable automated lung disease diagnosis in clinical practice.

## 1. Introduction

Lung diseases such as Idiopathic Pulmonary Fibrosis (IPF) and other forms of Interstitial Lung Disease (ILD) present significant diagnostic challenges due to overlapping radiological features and heterogeneous disease manifestations. High-Resolution Computed Tomography (HRCT) remains the primary imaging modality for ILD assessment, providing detailed visualization of lung tissue patterns such as Honeycombing and Ground Glass Opacity (GGO), which are important indicators of disease progression [1]. However, accurate differentiation of these patterns remains difficult even for experienced radiologists due to subtle textural and structural similarities, which may lead to diagnostic variability and potential misclassification in clinical interpretation [2]. Over the past decade, Deep Learning (DL) techniques have gained considerable attention in medical image analysis due to their ability to automatically extract hierarchical features and recognise complex patterns [3,4,5]. Convolutional Neural Networks (CNNs) and transfer learning-based architectures have demonstrated improved performance in pulmonary CT classification tasks compared to traditional machine learning methods, particularly in capturing fine-grained imaging features that are difficult to represent using handcrafted descriptors [6,7]. Deep learning frameworks, particularly Convolutional Neural Network (CNN)-based architectures, are widely adopted in lung image analysis pipelines for feature learning and classification tasks [8,9,10,11]. These approaches have been widely applied in lung cancer detection and COVID-19 classification [12,13], achieving promising results in sensitivity and generalisation performance.

Despite these advances, several methodological limitations persist in existing studies. Many works rely on small, institution-specific, or non-diverse datasets, which limit model generalisability across different populations and imaging protocols [14]. In addition, evaluating lung cancer detection models using multiple performance metrics is important, as accuracy alone may not fully reflect diagnostic performance [15]. Another key challenge lies in effectively capturing subtle radiological features required to distinguish Honeycombing from GGO. Although DL models offer strong feature-learning capabilities, their performance remains highly dependent on preprocessing quality, dataset variability, and training strategies [1].

Recent advancements in CT image preprocessing have shown that enhancement and standardisation techniques significantly influence DL performance. Denoising methods for low-dose CT imaging improve feature clarity while preserving anatomical structures, thereby enhancing classification reliability [16]. Similarly, Contrast Limited Adaptive Histogram Equalisation (CLAHE)-enhanced imaging improves local contrast and highlights subtle lung textures, which are essential for distinguishing fine-grained patterns such as Honeycombing and GGO [17,18]. Furthermore, deep learning-based lung segmentation pipelines improve classification performance by isolating relevant lung regions and reducing interference from surrounding anatomical structures [19]. These findings emphasise the importance of preprocessing in improving model robustness, particularly in ILD classification tasks involving subtle texture variations.

Although DL-based lung disease classification has advanced significantly, relatively fewer studies have focused specifically on Honeycombing pattern recognition within ILD. Existing reviews tend to provide broad overviews of pulmonary image analysis or focus on individual diseases without critically synthesising methodological limitations or identifying consistent gaps related to Honeycombing classification. This highlights the need for a more focused systematic synthesis of existing approaches to better understand current limitations and future research directions.

This systematic review aims to address this gap by providing a comprehensive evaluation of recent deep learning approaches for Interstitial Lung Disease (ILD)-related lung classification, with particular focus on honeycombing patterns. By systematically analysing existing methodologies, this study highlights the strengths and limitations of current approaches in distinguishing visually similar lung conditions. The review further synthesises key findings from previous studies to identify common challenges, including feature overlap, dataset variability, and misclassification issues. Overall, this work contributes to a better understanding of current deep learning developments in ILD diagnosis and identifies important directions for improving the reliability and clinical applicability of automated lung disease classification systems.

This study investigates the following research questions:

- RQ1: What deep learning architectures have been applied for honeycombing and ILD lung classification using CT images;
- RQ2: What datasets, preprocessing techniques, and feature extraction strategies are commonly used in existing studies;
- RQ3: What evaluation metrics are applied to assess model performance and how consistently they are reported;
- RQ4: What methodological limitations and challenges exist in current deep learning approaches for honeycombing classification;
- RQ5: What research gaps and future directions can be identified to improve automated ILD diagnosis;

The paper is structured as follows. Section 2 presents the materials and methods used in systematic review, including the online databases, PRISMA study selection process, medical image modalities, evaluation of dataset characteristics, image preparation methods, and classification performance metrics. Section 3 discusses the limitations of existing works in lung disease classification, covering limitations in classification models as well as the application of machine learning and deep learning approaches for lung classification. Section 4 presents the discussion and conclusion of the review, synthesizing the main findings and research gaps identified across the reviewed studies. Section 5 highlights future research directions and potential strategies to improve the robustness, generalisability, and clinical applicability of deep learning-based lung disease classification frameworks.

## 2. Materials and Methods

A comprehensive review was undertaken by systematically analysing studies obtained from eight distinct academic repositories. The primary focus was on Machine Learning (ML) and Deep Learning (DL)-based approaches for lung disease classification using Computed Tomography (CT) imaging, with particular attention to honeycombing and Ground Glass Opacity (GGO) patterns associated with interstitial lung diseases. The review also considered challenges in accurately distinguishing these visually similar patterns. To ensure a structured and thorough analysis, the review was organized around several key methodological aspects, including medical imaging modalities, dataset characteristics, image preprocessing techniques, and performance evaluation metrics.

The literature search was conducted using the following academic databases: IEEE Xplore, ScienceDirect, SpringerLink, PubMed, Scopus, Web of Science, ACM Digital Library, MDPI and Google Scholar. The search was restricted to studies published between 2011 and 2026 to capture recent developments in machine learning and deep learning-based medical image analysis. A structured search strategy was adopted using Boolean operators and three main components, as summarized in Table 1. This structured formulation ensured comprehensive coverage of relevant studies across disease-specific terms, learning-based methods, and CT image classification applications.

A combination of keywords and Boolean operators was used to retrieve relevant studies. The primary search query was structured into three main components: (i) *disease-related terms*, (ii) *deep learning techniques*, and (iii) *application context*.

The first component, (*“honeycombing lung” OR “interstitial lung disease” OR “ground glass opacity” OR “ILD”*), was used to capture studies related to the lung diseases of interest. The second component, *(“deep learning” OR “convolutional neural network” OR “CNN” OR “transfer learning”*), ensured the inclusion of studies applying advanced machine learning techniques.

The third component, *(“CT image classification” OR “medical image classification” OR “lung disease detection”*), was included to refine the search towards studies focusing on image-based classification and diagnostic applications. This component ensures that the retrieved studies are relevant to classification tasks within medical imaging, particularly those aligned with diagnostic workflows in clinical practice. Overall, this structured query design ensures that the selected studies are not only technically relevant but also aligned with real-world clinical applications, where automated image classification can support disease detection and assist clinical decision-making. Based on this structure, the final search query was formulated as follows:

*(“honeycombing lung” OR “interstitial lung disease” OR “ground glass opacity” OR “ILD”) AND (“deep learning” OR “convolutional neural network” OR “CNN” OR “transfer learning”) AND (“CT image classification” OR “medical image classification” OR “lung disease detection”).*

This component was included to ensure that the retrieved studies reflect real-world clinical imaging workflows, where CT scans are interpreted and used to support diagnostic decision-making. Specifically, it captures studies focusing on AI-assisted CT image classification for lung disease detection, aligning with radiological practice in diagnosing conditions such as interstitial lung disease, honeycombing patterns, and ground-glass opacity. This strengthens the clinical relevance of the search strategy by ensuring inclusion of studies applicable to diagnostic workflows in radiology.

Additional studies were identified through reference screening of selected articles to ensure coverage of relevant research. Study selection followed the Preferred Reporting Items for Systematic Reviews and Meta-Analyses (PRISMA) guidelines. Initially, all retrieved records from the selected databases were combined and duplicate studies were removed. Titles and abstracts were then screened to determine relevance. Studies that met the preliminary criteria were further evaluated through full-text review to confirm eligibility. The final set of studies included in this review comprised 98 articles focusing on machine learning and deep learning approaches for lung disease classification using CT images.

The inclusion criteria for selecting studies were defined as follows. First, studies must focus on machine learning or deep learning approaches for lung disease classification using CT images. Second, the research must involve interstitial lung disease patterns such as honeycombing or ground glass opacity. Third, studies must report experimental evaluation using performance metrics such as accuracy, sensitivity, specificity, precision, F1-score, or AUC-ROC. Finally, only peer-reviewed journal articles and conference papers published in English between 2011 and 2026 were considered.

The exclusion criteria were also applied to ensure the relevance and quality of the selected studies. Studies focusing on non-CT imaging modalities such as X-ray or ultrasound were excluded unless CT imaging formed a core component of the analysis. Review papers, editorials, theses, and non-peer-reviewed publications were also excluded. In addition, studies that did not provide sufficient methodological or experimental details, or those that focused on unrelated lung diseases without relevance to interstitial lung disease patterns, were removed from the review.

The selected studies were examined to identify CT image acquisition and processing methods, dataset size and characteristics, and the extent of class imbalance among normal, honeycombing, and ground glass opacity cases. Image preprocessing techniques, including normalization, augmentation, noise reduction, and Region of Interest (ROI) extraction, were analysed to determine their impact on classification performance. Additionally, commonly used evaluation metrics such as accuracy, precision, sensitivity, specificity, and Area Under the Receiver Operating Characteristic Curve (AUC-ROC) were compared to highlight the strengths and limitations of different approaches. These methodological components are further discussed in the following sections.

### 2.1. PRISMA Study Selection Process

Study selection followed the Preferred Reporting Items for Systematic Reviews and Meta-Analyses (PRISMA 2020) guidelines to ensure comprehensive coverage of relevant research and is summarized in Figure 1. The process consisted of four sequential stages. (i) *Identification*: records were retrieved from eight academic databases using the defined search queries (*n* = 750), and an additional 28 records were identified through reference-list screening of relevant articles. A total of 468 unique records remained after duplicate removal and were subsequently subjected to title and abstract screening. During this stage, 120 records were excluded based on the predefined inclusion and exclusion criteria. (ii) *Screening*: the titles and abstracts of the 468 records were screened against the predefined inclusion and exclusion criteria. At this stage, 120 records were excluded because they focused on non-CT imaging modalities, were not related to lung disease classification, did not employ machine learning or deep learning techniques, were review or editorial articles, or were not published in English. (iii) *Eligibility*: the remaining 348 articles underwent full-text assessment to determine their suitability for inclusion. A further 250 articles were excluded due to insufficient methodological details, lack of quantitative performance metrics, focus on lung conditions outside the scope of honeycombing, ground glass opacity, or interstitial lung disease, use of CT imaging as a secondary modality, or duplication of previously reported analyses. (iv) *Inclusion*: the final set of 98 studies was included in the qualitative synthesis. The comparative analyses of representative studies according to their respective methodological categories form the basis of the discussion and synthesis presented throughout this review.

Figure 1 presents the PRISMA 2020 flow diagram of the study selection process. Of the 468 unique records that entered the screening stage, 120 were excluded from the following title and abstract screening. The remaining 348 studies underwent full-text assessment, from which 250 articles were excluded based on the predefined eligibility criteria. Consequently, 98 studies satisfied all inclusion requirements and were included in the qualitative synthesis. These studies formed the foundation for the comparative analysis of imaging modalities, datasets, preprocessing techniques, deep learning architectures, and evaluation metrics discussed in this review.

### 2.2. Medical Image Modalities

Medical imaging modalities play an essential role in lung disease diagnosis by providing different levels of anatomical, structural, and functional information. The selection of an appropriate imaging modality is an important consideration in developing automated lung disease classification systems because differences in image characteristics, resolution, acquisition methods, and clinical application can influence the quality of extracted features and model performance. This review considers four major imaging modalities commonly applied in lung disease analysis: X-ray, Computed Tomography (CT), Magnetic Resonance Imaging (MRI), and Positron Emission Tomography (PET). Among these modalities, CT imaging provides detailed cross-sectional visualization of lung structures with high spatial resolution, making it suitable for analysing complex pulmonary patterns and abnormalities. The availability of detailed anatomical information from CT images has contributed to its extensive application in computer-aided lung disease classification studies [20,21,22,23,24]. However, CT images may contain variations caused by acquisition settings, noise, and imaging protocols, requiring appropriate preprocessing strategies to improve image consistency and feature representation [9,25].

X-ray imaging remains an important imaging modality due to its accessibility, low cost, and suitability for large-scale screening applications. However, its two-dimensional projection may limit the visualization of subtle structural abnormalities compared with three-dimensional imaging modalities. MRI offers excellent soft tissue contrast and provides additional information for selected pulmonary conditions, although its application in lung imaging is constrained by technical challenges such as low proton density and respiratory motion artifacts [26,27]. PET imaging primarily focuses on functional and metabolic information, providing complementary insights beyond structural imaging.

Table 2 summarizes the contribution of different medical imaging modalities reported in lung disease diagnosis and classification studies. The findings indicate that CT is the most frequently adopted modality among the representative studies, particularly for applications involving automated classification of pulmonary abnormalities [28,29]. This preference is mainly associated with the ability of CT to provide detailed visualization of lung parenchymal structures, which supports the identification of complex patterns such as honeycombing and ground-glass opacity.

In comparison, X-ray-based approaches remain valuable for rapid screening and classification tasks due to their availability and cost-effectiveness. However, the limited dimensional information provided by X-ray images may restrict the detection of subtle abnormalities. MRI-based studies represent a smaller proportion of lung classification research but demonstrate potential advantages in specific applications requiring enhanced soft tissue characterization. PET, although less commonly applied in automated lung pattern classification, provides functional information that may complement structural imaging approaches. Overall, the comparison in Table 2 highlights that the choice of imaging modality is influenced by the clinical objective, disease characteristics, and requirements of automated classification systems. The predominance of CT-based approaches in the reviewed studies suggests that detailed structural information remains a key factor in developing reliable deep learning-based lung disease classification models.

### 2.3. Evaluation of Dataset Characteristics

In lung disease image analysis studies, datasets are generally categorized into public datasets and private clinical datasets based on their source, accessibility, and data collection strategy. Public datasets are openly available repositories that can be accessed by researchers worldwide, often through research consortium databases or open-access platforms. These datasets support reproducible research and enable comparative evaluation across different approaches. However, variations in annotation protocols, labeling consistency, and clinical validation procedures may affect dataset reliability across studies.

In contrast, private or clinical datasets are collected from hospitals or medical institutions under controlled conditions, often involving collaboration with medical experts. These datasets may provide more clinically specific information and expert-validated annotations; however, access is commonly restricted due to patient privacy regulations and institutional policies. Consequently, limited availability of clinical datasets may reduce external validation and benchmarking across different studies. The distinction between public and clinical datasets is important in lung disease image analysis, as dataset characteristics directly influence model performance, generalization capability, and clinical applicability. Table 3 presents a comparative summary of dataset size, class distribution, annotation practices, database collection strategies, and image modalities used in recent lung disease classification and segmentation studies. A notable limitation observed across several studies is the insufficient reporting of dataset characteristics, particularly dataset size and class distribution, which may reduce transparency, reproducibility, and meaningful comparison between different approaches.

Class imbalance is frequently observed across lung disease datasets, particularly when abnormal findings such as honeycombing, ground glass opacity, and malignant lung nodules are compared with normal or benign categories. Public datasets, such as those reported in [31,32,34], facilitate open-access research and enable reproducible comparison across different studies. For example, the pneumonia dataset reported in [34] was obtained from the Kaggle public repository and consisted of 6000 pre-labelled chest X-ray images. Similarly, studies [31,32] employed the publicly available LIDC-IDRI database, which contains CT images annotated by multiple radiologists for lung nodule analysis. These publicly available datasets provide valuable benchmark resources; however, differences in annotation protocols, labeling criteria, and preprocessing strategies may introduce variability across studies.

In contrast, clinical datasets reported in [30,35] were collected directly from medical institutions with radiologist-annotated or radiologist-verified data. Such datasets generally provide clinically reliable labels and expert interpretation, thereby improving the quality of the ground truth. For example, study [30] utilized 14,696 CT image patches (regions of interest) extracted from high-resolution CT scans, where the lung tissue patterns were annotated by radiologists. Similarly, study [35] employed a clinical CT dataset containing 1218 examinations with radiologist-verified abnormal lung regions, supporting both segmentation and classification tasks. Although clinical datasets often provide higher annotation reliability, their restricted accessibility and relatively limited size reduce reproducibility and external benchmarking.

Several studies addressed dataset imbalance using preprocessing and augmentation techniques. Study [31] initially utilized 776 annotated lung nodules from the LIDC-IDRI database, comprising 186 benign and 590 malignant nodules, resulting in a highly imbalanced dataset. Data augmentation was subsequently applied to expand the dataset to 14,520 samples, including 7440 benign and 7080 malignant samples, producing a substantially more balanced dataset for model training. In comparison, study [32] employed 8474 CT images from the same LIDC-IDRI database, consisting of 3259 benign and 5215 malignant lung nodules. Although the dataset remained imbalanced, the larger sample size provided greater variability for model learning. These findings demonstrate that augmentation and dataset expansion can improve class representation, thereby reducing model bias towards majority classes and enhancing classification performance.

Segmentation-focused datasets, such as those reported in [33,35], provide localized region information that supports learning of abnormal lung structures. However, these datasets are often limited in scope and may not fully represent the diversity of lung disease patterns required for robust classification models. Furthermore, variations in scanner type, acquisition settings, annotation procedures, and radiologist interpretation introduce heterogeneity in image quality and ground truth labels, which may negatively affect model generalization and cross-dataset performance.

Overall, the analysis demonstrates that dataset quality is determined not only by dataset size but also by annotation reliability, class distribution, preprocessing strategy, and data collection methodology. The findings in Table 3 indicate that differences in model performance are influenced not only by algorithm design but also by dataset characteristics. Therefore, improving annotation standardization, increasing multi-institutional data sharing, and developing balanced, well-annotated datasets are essential for building robust and clinically reliable lung disease image analysis systems.

### 2.4. Image Preparation Methods

Image preparation represents an essential stage in medical image analysis, particularly for deep learning-based lung disease classification and related diagnostic tasks. These processes aim to improve image consistency, enhance important visual characteristics, reduce irrelevant information, and provide more informative inputs for feature extraction. The reviewed studies reported various image preparation strategies, including image augmentation, normalization, scaling, Region of Interest (ROI) extraction, segmentation-based approaches, and image enhancement techniques. These approaches contribute to improving model generalization, reducing overfitting, and increasing feature consistency across heterogeneous medical imaging datasets. Although several image preparation strategies were specifically developed and evaluated for lung imaging applications, some techniques were also reported across different medical imaging modalities, such as brain MRI. These approaches remain relevant because similar challenges, including limited dataset size, image variability, noise, and inconsistent acquisition conditions, are commonly encountered across medical imaging applications. Normalization is widely applied to standardize pixel intensity distributions and reduce variations caused by differences in scanners, acquisition protocols, and imaging conditions. For lung CT applications, normalization methods improve input consistency and support stable deep learning model training by reducing intensity variation and scanner-dependent artefacts [36,37,38]. These approaches allow models to focus more effectively on disease-related characteristics rather than acquisition-specific variations. Similar normalization strategies have also been investigated in multi-site medical imaging environments to improve robustness and cross-dataset performance.

Image augmentation is another commonly adopted strategy in medical imaging, particularly when available datasets are limited. It involves generating additional training variations through geometric and intensity-based transformations, including rotation, flipping, and noise-based augmentation. In studies involving lung image analysis, augmentation techniques have been applied to increase dataset diversity and improve model robustness during training [31,39]. Furthermore, augmentation strategies have also been widely explored across radiology and brain MRI applications, demonstrating their ability to reduce overfitting and improve generalization capability [40,41,42].

ROI extraction focuses on identifying and isolating clinically meaningful regions while reducing irrelevant background information. Depending on the application, ROI generation may involve patch extraction, thresholding, morphological operations, or localization-based techniques. For lung imaging studies, ROI-based approaches have been applied to highlight abnormal lung regions, nodules, and disease-related structures before classification or further analysis [16,35,43,44]. Similar ROI-based strategies have also been adopted across different medical imaging modalities to improve feature representation by directing deep learning models toward relevant anatomical regions [41]. Image scaling is another important preparation step, particularly for deep learning architectures that require fixed input dimensions. Images obtained from different datasets may vary in resolution and spatial characteristics; therefore, resizing using interpolation-based methods ensures consistent input representation while preserving important structural information. Several medical imaging studies have applied scaling and resizing strategies to standardize image inputs before model training [37,39,45,46].

Segmentation-based image preparation is also frequently employed in medical image analysis to separate anatomical structures or pathological regions before classification. Unlike ROI extraction, which focuses on selecting relevant regions, segmentation provides more precise boundaries between target structures and surrounding areas. Within lung image analysis, segmentation approaches have been used to extract lung fields, lung parenchyma, and abnormal regions, allowing deep learning models to focus on disease-related information while reducing irrelevant anatomical interference [16,19,45,46,47]. Although segmentation can also represent a primary analysis task, it is commonly integrated as an image preparation step to improve downstream classification and feature learning. Table 4 summarizes the image preparation methods identified across the reviewed studies. The table presents the methodological approaches, their advantages, and representative studies demonstrating how different preparation strategies contribute to improving image quality, feature representation, and deep learning model robustness. The included examples cover lung-specific applications as well as representative image preparation approaches reported across different medical imaging modalities.

In addition to the conventional image preparation approaches summarized in Table 4, several studies have highlighted the importance of advanced enhancement and refinement techniques for improving medical image analysis performance. Contrast Limited Adaptive Histogram Equalization (CLAHE) is an enhancement technique that improves local contrast and increases the visibility of subtle structures. For lung imaging applications, CLAHE-based approaches have been explored to enhance abnormal patterns and improve feature extraction from medical images [16,17]. Segmentation-based preparation remains particularly important in lung image analysis because accurate isolation of lung structures and pathological regions can reduce irrelevant information and improve model focus. Studies involving lung field, lung parenchyma, and pathological region segmentation demonstrate that segmentation-based inputs can support more effective downstream classification and analysis [16,19,45,46,47].

Furthermore, deep learning-based denoising techniques have gained increasing attention, particularly in low-dose CT imaging, where reducing noise while maintaining diagnostic information is essential. These approaches improve image quality by suppressing unwanted artefacts and may enhance the robustness of deep learning models through cleaner input representations [18]. Overall, the findings summarized in Table 4 demonstrate that image preparation strategies significantly influence the accuracy, stability, and generalization capability of deep learning models for lung disease classification and broader medical image analysis. However, many existing studies still rely mainly on conventional preparation pipelines and do not fully exploit advanced strategies such as CLAHE enhancement, segmentation-based localization, and deep learning-based denoising.

Future development of robust lung disease classification systems requires improved standardization of image preparation procedures, integration of clinically meaningful regions, and adoption of advanced enhancement strategies. These improvements are particularly important for reducing misclassification between visually similar lung conditions, such as honeycombing and Ground Glass Opacity (GGO), where subtle image characteristics strongly influence model predictions.

### 2.5. Classification Performance Metrics

Evaluation metrics are essential for assessing the performance of deep learning-based lung disease classification systems. After preprocessing, training, and validation, test images are evaluated by comparing predicted labels against ground truth annotations using the confusion matrix. True Positive (TP) and True Negative (TN) represent correctly classified cases, whereas False Positive (FP) and False Negative (FN) represent classification errors. From a clinical perspective, False Negatives (FN) are particularly critical in lung disease classification, including conditions involving honeycombing and interstitial lung abnormalities, as they represent missed abnormal cases that may affect timely clinical assessment. In contrast, False Positives (FP) may result in unnecessary follow-up examinations and increased clinical workload. Therefore, sensitivity is important for assessing the ability of a model to correctly identify abnormal cases, while specificity evaluates the ability to correctly identify non-diseased cases and reduce false alarms. However, accuracy alone may be insufficient to fully evaluate model performance in medical imaging tasks, particularly when datasets involve imbalanced classes or heterogeneous distributions. In such situations, high accuracy may be achieved by correctly classifying dominant classes while failing to detect minority or clinically significant cases. Therefore, the use of multiple evaluation metrics, including precision, sensitivity, specificity, and AUC-ROC, provides a more comprehensive assessment of model performance. Table 5 summarizes the evaluation metrics reported in representative lung disease classification studies among the included literature.

Table 5 presents a summary of evaluation metrics reported in representative lung disease classification studies. The findings indicate that accuracy is the most consistently reported metric, with all selected studies including accuracy as part of their performance evaluation. This demonstrates that accuracy remains the primary measure used for assessing classification performance, mainly because it provides a straightforward indication of the proportion of correctly classified cases. However, accuracy alone may not sufficiently represent model effectiveness in medical imaging applications, particularly when the consequences of misclassification differ between abnormal and normal cases.

Sensitivity was frequently reported among the reviewed studies, reflecting the importance of measuring the ability of models to correctly identify abnormal lung cases. In lung disease classification, high sensitivity is essential because missed abnormal cases may result in delayed clinical assessment. Therefore, sensitivity provides additional insight into model performance by focusing on the detection of disease-related patterns rather than overall classification correctness alone. In contrast, specificity was reported less consistently across the selected studies. This variation may be attributed to differences in evaluation priorities, where some studies primarily emphasize disease detection performance rather than the ability to correctly identify non-diseased cases. However, the absence of specificity reporting limits the assessment of false positive errors, which may influence the reliability of automated classification systems in clinical settings. Precision and AUC-ROC were also reported with varying frequency among the reviewed studies, indicating differences in the comprehensiveness of evaluation frameworks. Precision provides additional information regarding the reliability of positive predictions, while AUC-ROC evaluates the discriminative capability of classification models across different decision thresholds. The variation in reported metrics suggests that a standardized evaluation framework has not yet been consistently adopted across lung disease classification studies.

Several studies, including [50,51,52,56,57,58,60], incorporated multiple evaluation metrics involving accuracy, precision, sensitivity, specificity, and AUC-ROC, enabling a more comprehensive assessment of model performance. In contrast, studies reporting fewer metrics provide limited insight into specific error patterns and classification reliability. Overall, Table 5 highlights that although accuracy remains the dominant evaluation measure, the adoption of multi-metric evaluation frameworks is necessary to support fair comparison and improve the reliability of deep learning-based lung disease classification systems.

## 3. Limitations of Existing Works

### 3.1. Limitations in Classification Models

Lung disease classification has primarily been approached using two main computational paradigms: traditional Machine Learning (ML) and Deep Learning (DL). Both approaches have contributed significantly to medical image analysis; however, each exhibits inherent limitations that affect diagnostic accuracy and model generalisation, particularly in complex cases such as Honeycombing (HC) and Ground Glass Opacity (GGO).

#### 3.1.1. Machine Learning for Lung Classification

Machine learning has been widely employed in medical image analysis to enable systems to learn patterns from data without relying on explicit rule-based programming. In lung imaging analysis, Machine Learning (ML) algorithms extract discriminative features that assist in identifying structural abnormalities associated with pulmonary diseases, thereby improving diagnostic consistency and reducing inter-observer variability [2]. However, the effectiveness of these approaches is highly dependent on dataset quality, feature representation, and class distribution. Traditional ML methods commonly rely on handcrafted feature extraction, involving the manual selection of texture-based, shape-based, and intensity-based descriptors. This dependence may limit scalability, adaptability, and robustness when applied to large or heterogeneous clinical datasets. Consequently, ML performance can vary significantly across studies, particularly for complex lung conditions such as Honeycombing and Ground Glass Opacity. Table 6 presents a comparative summary of representative machine learning models applied for lung disease classification, highlighting the targeted pulmonary conditions and their corresponding performance metrics.

The findings summarized in Table 6 indicate that Support Vector Machine (SVM)-based approaches and Bayesian classifiers demonstrate promising performance among traditional machine learning methods, particularly for Diffuse Interstitial Lung Disease (DILD) and diffuse lung pattern classification. In [2], a context-sensitive Support Vector Machine (csSVM) was proposed for HRCT-based DILD pattern classification. Unlike conventional SVM, the proposed csSVM incorporated regional contextual information from neighbouring lung regions to improve classification performance. The csSVM achieved an accuracy of 89.88%, compared with 87.39% obtained by the conventional SVM for ROI-based classification. Similarly, Ref. [64] evaluated SVM and Bayesian classifiers for HRCT-based DILD pattern classification with the objective of reducing interscanner variation. The SVM classifier demonstrated superior performance, achieving an accuracy of 92%, whereas the Bayesian classifier achieved an accuracy of 77% under the reported evaluation condition. These findings demonstrate that traditional machine learning classifiers can effectively distinguish lung disease patterns when appropriate feature representations are available. However, their performance remains dependent on handcrafted feature extraction, which may limit their adaptability when applied to complex medical images containing subtle texture variations and heterogeneous lung abnormalities. K-Nearest Neighbour (KNN) classifiers have also been applied in various pulmonary disease classification tasks, including tuberculosis and multi-class pulmonary abnormality classification [67,69]. The reported accuracy values range from 71.81% to 94%, indicating that KNN performance is strongly influenced by feature representation, distance measurement, and dataset characteristics. In addition, Ref. [65] evaluated KNN together with other machine learning classifiers, including Support Vector Machine (SVM), Random Forest (RF), and Decision Tree (DT), for CT-based lung cancer detection. The study classified lung CT images into malignant lung cancer and normal categories using Gray Level Co-occurrence Matrix (GLCM)-based texture features. Among the evaluated classifiers, SVM achieved the highest accuracy of 99.32%, with precision, recall, and F1-score values of 99%, demonstrating the effectiveness of handcrafted texture features for distinguishing lung cancer patterns. Although KNN provides a simple and interpretable classification strategy, its sensitivity to feature scaling, noise, and variations in data distribution may reduce its robustness when applied to heterogeneous clinical imaging datasets.

To overcome the limitations of conventional machine learning methods, hybrid and optimized approaches have also been explored. A hybrid SVM and Feed-Forward Backpropagation Neural Network (FFBPNN) model demonstrated improved lung cancer classification performance, achieving an accuracy of 98.08%, precision of 98.17%, recall of 96.50%, and F1-score of 97.00% [66]. Similarly, an enhanced KNN classifier integrated with a hybrid feature selection methodology improved COVID-19 detection by selecting more discriminative features before classification, achieving an accuracy of 96% [68]. These findings highlight that feature optimization and hybrid learning strategies can enhance the predictive capability of traditional machine learning models by improving feature representation and classification performance.

Furthermore, Multiple Instance Learning (MIL) combined with Active Learning (AL) has been investigated for tuberculosis detection, achieving an AUC value of 0.71 [56]. The integration of Active Learning provides an advantage by reducing dependency on extensive manual annotation through the selection of more informative samples for model training. Nevertheless, similar to other machine learning approaches, MIL-based methods remain dependent on feature quality and the availability of labelled data.

Overall, Table 6 demonstrates that traditional machine learning approaches, including SVM, Bayesian classifiers, KNN, hybrid models, and MIL-based strategies, can achieve promising performance in lung disease classification. However, these approaches are generally constrained by their reliance on handcrafted feature extraction and limited capability to automatically learn complex and hierarchical image representations. These limitations motivate the adoption of deep learning approaches, which are discussed in the following section.

#### 3.1.2. Deep Learning for Lung Classification

Deep Learning (DL), a subset of machine learning, employs multilayer artificial neural networks to perform hierarchical features learning directly from raw data. Unlike traditional machine learning classifiers such as Support Vector Machine (SVM), K-Nearest Neighbour (KNN), Artificial Neural Networks (ANN), and Probabilistic Neural Networks (PNN), DL automatically learns discriminative feature representations through backpropagation and iterative optimization. This capability enables DL models to capture complex spatial, structural, and textural patterns in medical images, particularly in CT and chest X-ray imaging.

Convolutional Neural Networks (CNNs) and transfer learning-based architectures have demonstrated strong performance in identifying pulmonary abnormalities, including pneumonia, COVID-19, lung cancer, honeycombing, and Ground-Glass Opacity (GGO) patterns [70,71]. Their ability to automatically learn hierarchical feature representations has contributed to significant improvements in classification accuracy and robustness across diverse medical imaging datasets.

Table 7 presents a comparative summary of existing deep learning approaches for lung disease classification, highlighting the adopted architectures, target disease categories, and reported performance metrics. The reviewed studies include a wide range of CNN-based and transfer learning-based models, including VGG16, ResNet variants, DenseNet, and EfficientNet, applied to both CT and chest X-ray datasets. These studies cover diverse lung conditions, including normal cases, pneumonia, COVID-19, lung cancer, and interstitial lung diseases, reflecting substantial variation in dataset design, imaging modality, and classification objectives across literature.

As shown in Table 7, a comparative summary of deep learning models for lung disease classification is presented, including their architectures and corresponding performance metrics reported in previous studies.

In lung disease classification, CNN-based models have been widely explored. In [6], a CNN-based technique was proposed for COVID-19 CT scan classification, demonstrating the effectiveness of deep learning in identifying COVID-19-related abnormalities. Studies such as [76,77,78] further demonstrate the effectiveness of VGG16-based and hybrid deep learning architectures for lung disease and lung cancer-related classification tasks. In [76], a VGG16-based lung disease classification approach integrated with Principal Component Analysis (PCA) was proposed for feature reduction. The method was evaluated using the NIH ChestX-ray14 dataset containing multiple thoracic disease categories and achieved an accuracy of 96%, demonstrating the potential of combining deep feature extraction with dimensionality reduction techniques for thoracic disease classification. Meanwhile, Ref. [77] evaluated several deep learning architectures, including VGG16, ResNet50, InceptionV3, and MobileNetV2, for three-class lung cancer classification using CT images. The study classified the images into Normal, Benign, and Malignant categories. Among the evaluated architectures, VGG16 achieved the highest test accuracy of 98.18%, followed by ResNet50 at 93.33% and InceptionV3 at 88.79%. Although MobileNetV2 achieved the highest training accuracy of 97.15%, its training performance cannot be directly compared with the test accuracy of the other models. Overall, VGG16 demonstrated the strongest reported test performance among the evaluated architectures; however, the relatively small dataset may limit the generalizability of the findings to unseen data. Similarly, Ref. [78] proposed a two-step approach for lung cancer assessment, where a Decision Tree classifier first evaluated the patient’s risk level based on symptoms and medical-background information, achieving an accuracy of 99.67%. For patients classified as medium or high risk, a customized VGG16 CNN was subsequently applied to CT scan images to identify the presence and type of lung cancer, achieving an accuracy of 92.53%. The findings demonstrate the potential of combining conventional machine learning-based risk assessment with deep learning-based CT image analysis in a sequential diagnostic framework. However, the lower accuracy of the VGG16 CNN compared with the Decision Tree indicates that CT-based classification remained more challenging than the initial risk assessment. The authors also identified the need for larger training datasets to reduce overfitting and improve generalization, while suggesting newer architectures such as ResNet and InceptionNet as potential alternatives for improving CNN performance. However, despite the high classification performance reported by these CNN-based approaches, challenges remain when applying such models to other complex lung abnormalities, particularly visually similar patterns such as ground-glass opacity (GGO) and honeycombing. These patterns exhibit subtle variations in texture, intensity, and spatial characteristics, which may increase the risk of misclassification. This limitation was also reported in [33], where a CNN-based approach was used to classify healthy lungs, ground-glass opacity (GGO), and honeycombing from CT images. The results showed variations in classification performance across different lung texture categories, with lower accuracy for GGO (73.3%) and honeycombing (69.1%) compared with healthy lungs (87.8%), highlighting the difficulty of distinguishing subtle interstitial lung patterns. Similarly, Ref. [39] addressed the challenge of limited HRCT training samples by introducing a Perlin noise-based data augmentation strategy, demonstrating that increasing dataset diversity can improve deep learning model robustness and generalization. To further improve feature extraction capability and classification performance, subsequent studies have investigated deeper and more advanced convolutional architectures, including residual and attention-based networks. ResNet-based architectures have been widely adopted in medical image classification due to their residual learning capability, which enables deeper feature extraction while reducing vanishing gradient problems. In [79], a modified ResNet-50 model was proposed for lung cancer detection and achieved an accuracy of 98.77%, demonstrating the effectiveness of residual architectures in extracting discriminative features from medical images. In contrast, Ref. [81] explored an attention-based EfficientNet architecture for lung disease detection using lung sound signals, demonstrating that attention mechanisms combined with pre-trained deep learning models can enhance feature representation for respiratory disease classification. Studies reported in [33] investigated pneumonia detection in chest X-ray images using deep learning architectures. The study developed an improved hybrid model based on the pre-trained ResNet50 architecture and compared its performance with DenseNet201, ResNet50, InceptionV3, GoogleNet, and AlexNet. The improved model achieved the highest accuracy (97.12%), specificity (97.69%), and F1-score (94.51%) among the evaluated models, with a sensitivity of 95.78%. Compared with the standard ResNet50, which achieved an accuracy of 96.35%, sensitivity of 92.91%, specificity of 97.54%, and F1-score of 92.91%, the improved model showed higher performance across all four reported metrics. However, AlexNet achieved the highest sensitivity (98.34%), although its specificity (89.54%) and F1-score (79.28%) were considerably lower. InceptionV3 achieved the same specificity as the improved model (97.69%) but a slightly lower F1-score (94.33%). Overall, the improved ResNet50-based model demonstrated the strongest overall performance among the evaluated models, particularly in terms of accuracy, specificity, and F1-score. However, performance variations may occur when deep learning models are applied to small, heterogeneous, or disease-specific datasets. For example, COVID-19 and lung disease classification studies have reported variations in performance due to differences in dataset characteristics, image patterns, and disease complexity [6,82]. These factors may affect the ability of models to learn robust and generalizable features, particularly when medical images contain complex and visually similar abnormalities. Compared with earlier ResNet variants, ResNet50v2 incorporates improved residual block designs with pre-activation structures that can support more stable optimization and feature learning. A review by [72] summarized the application of ResNet variants, including ResNet-50, ResNet-101, and ResNet-152, for lung disease classification using chest X-ray images. The review highlighted the importance of residual learning strategies in improving feature representation for medical image classification tasks.

Beyond conventional CNN- and ResNet-based architectures, recent studies have explored alternative deep learning models to improve lung disease classification performance. DenseNet-based models have demonstrated strong performance through dense connectivity, which promotes feature reuse and improves gradient propagation across network layers. For example, Ref. [76] proposed an attention-based modified DenseNet201 model for classifying normal, pneumothorax, and atelectasis (pulmonary lung diseases) from chest X-ray images, achieving robust performance by enhancing feature representation through attention mechanisms. More recently, Ref. [17] employed CLAHE-enhanced CT images from the LIDC-IDRI dataset as a preprocessing strategy, combined with Swin Transformer-based feature extraction and XGBoost classification for benign and malignant lung nodule classification. The proposed hybrid pipeline achieved an accuracy of 95.8%, demonstrating the potential of transformer-based feature extraction combined with machine learning classifiers for capturing complex CT image characteristics. For instance, Ref. [49] combined EfficientNet-B7 with VGG16 for lung cancer classification using histopathological images. The hybrid architecture demonstrated the potential of integrating complementary feature extraction networks to enhance discriminative feature learning and achieved an accuracy of 98.73%. This finding suggests that combining different deep learning architectures can improve the capability of models to capture complex patterns associated with lung cancer classification. Comparative studies such as [58] evaluated VGG16, ResNet-50, and InceptionV3 for lung disease classification, demonstrating that different architectures exhibit varying performance depending on dataset characteristics, disease categories, and imaging variations. Beyond single-task classification, multi-task learning frameworks such as [73] jointly performed classification and segmentation of COVID-19 CT images, enabling shared feature learning across related tasks and improving the representation of disease-related patterns. In addition, Ref. [74] proposed a CNN-based model, named DarkCovidNet, for automated COVID-19 detection and classification from chest X-ray images. The model performed binary classification of COVID-19 and No-Findings (normal) cases and multi-class classification of COVID-19, No-Findings, and Pneumonia cases. For binary classification, DarkCovidNet achieved an accuracy of 98.08%, sensitivity of 95.13%, specificity of 95.30%, precision of 98.03%, and F1-score of 96.51%. For multi-class classification, the model achieved an accuracy of 87.02%, sensitivity of 85.35%, specificity of 92.18%, precision of 89.96%, and F1-score of 87.37%. The substantial reduction in performance from binary to multi-class classification indicates that distinguishing COVID-19 from both normal and pneumonia cases remains more challenging than binary detection. This suggests that although DarkCovidNet demonstrated strong performance in separating COVID-19 from normal cases, its classification performance was more limited when pneumonia was included as an additional class. Furthermore, Ref. [80] demonstrated the effectiveness of a VGG16-based architecture for histopathological lung disease classification. The study highlighted that appropriate preprocessing and fine-tuning strategies can enhance feature extraction capability and improve classification performance. These findings indicate that transfer learning-based approaches remain valuable for analysing complex medical images, particularly when domain-specific image characteristics need to be captured. The investigation in [58] evaluated transfer-learning-based deep learning models for lung disease classification using both non-segmented and segmented chest X-ray (CXR) images. Three pre-trained CNN models, namely VGG16, ResNet-50, and InceptionV3, were evaluated for classifying healthy, pneumonia, and tuberculosis cases, with a U-Net segmentation network used to extract the lung regions before classification. Among the evaluated models, the InceptionV3 model trained on segmented images achieved the best overall performance, with an accuracy of 82%. For the segmented InceptionV3 model, the AUC, F1-score, precision, and sensitivity were 90%, 76%, 75%, and 77%, respectively, for healthy cases; 99%, 93%, 90%, and 95%, respectively, for pneumonia; and 93%, 78%, 81%, and 75%, respectively, for tuberculosis. The higher performance for pneumonia and tuberculosis compared with healthy cases, particularly the AUC of 99% and sensitivity of 95% for pneumonia, indicates that the segmented InceptionV3 model was more effective in identifying diseased cases. The results also suggest that lung segmentation contributed to stronger classification performance, as the segmented InceptionV3 model achieved higher performance across most reported metrics than its non-segmented counterpart. However, the lower performance for healthy cases indicates that distinguishing normal lungs from diseased lungs remained more challenging. Overall, the study demonstrated that transfer learning combined with lung segmentation can achieve competitive classification performance despite the use of a relatively small dataset.

Although deep learning architectures such as CNN, ResNet variants, VGG16, DenseNet, EfficientNet, and transformer-based models have significantly improved lung disease classification performance [17,34,43,49,58,75,79,80,81,82] their effectiveness remains influenced by variations in dataset characteristics, image quality, annotation consistency, and the presence of complex pathological patterns 39,49,72,73]. Consequently, enhanced preprocessing techniques, effective data augmentation strategies, hybrid architectures, and multi-task learning frameworks have emerged as promising approaches to improve feature representation, model generalisation, and the reliability of computer-aided diagnosis systems in clinical applications [33,34,75,82,83].

## 4. Discussion and Conclusions

This study presented a comprehensive review of machine learning and deep learning approaches for lung disease classification using CT imaging, with particular emphasis on honeycombing and Ground-Glass Opacity (GGO) patterns associated with interstitial lung diseases. The review examined studies published between 2011 and 2026, focusing on imaging modalities, dataset characteristics, preprocessing techniques, deep learning architectures, and performance evaluation metrics. The findings indicate that CT imaging remains an effective modality for detecting lung abnormalities because of its high spatial resolution and ability to capture fine structural patterns such as fibrosis, honeycombing, and ground-glass opacity. Comparative analysis further indicates that deep learning approaches, particularly Convolutional Neural Networks (CNNs) and Residual Network (ResNet) architectures, generally demonstrated competitive or stronger classification performance than traditional machine learning methods across the reviewed studies. Transfer learning approaches also demonstrated strong performance by leveraging pretrained knowledge and adapting it to medical imaging tasks. However, the reviewed literature continues to exhibit limitations related to dataset size, class imbalance, annotation consistency, preprocessing strategies, and evaluation practices.

A primary research gap identified in this review is the limited emphasis on honeycombing lung classification. Existing research has predominantly focused on more extensively studied pulmonary conditions, including lung cancer, pneumonia, and COVID-19, whereas comparatively fewer studies have investigated the specific challenge of differentiating honeycombing from GGO. These patterns may exhibit subtle and overlapping visual characteristics in CT images, making accurate differentiation challenging and potentially increasing the likelihood of misclassification. This limitation is particularly important for interstitial lung disease classification because inaccurate identification of visually similar patterns may affect the reliability of automated diagnostic support. Another important gap concerns the evaluation of model performance. Many reviewed studies rely heavily on accuracy as the primary performance measure. Although accuracy provides an overall indication of classification performance, it may not adequately reflect diagnostic performance when datasets contain imbalanced disease classes. Sensitivity and specificity are particularly important in medical imaging because they provide complementary information regarding the identification of diseased and non-diseased cases. For multiclass classification, metrics such as One-vs-Rest Area Under the ROC Curve (OvR AUC), together with precision, recall, and F1-score, can provide a more comprehensive assessment of class-specific and overall model performance. The inconsistent reporting of these complementary metrics across studies therefore limits direct comparison and makes it difficult to determine the robustness of reported classification results.

Dataset-related limitations also remain a significant concern. Many studies rely on relatively small public datasets, which can support reproducibility but may contain variations in annotation quality, imaging protocols, scanner settings, and patient characteristics. Such variations may introduce noise and affect model robustness. Hospital-based datasets collected under clinical supervision may provide expert radiologist annotations and more reliable diagnostic labels, but these datasets are often limited in size and diversity because of ethical, privacy, and data-access requirements. Consequently, models trained using either public or institution-specific datasets may experience reduced performance when applied to external clinical populations or different imaging environments. The development and evaluation of models using diverse datasets are therefore important for establishing their generalizability and clinical reliability.

The review also identified insufficient investigation of domain-specific preprocessing techniques for lung CT classification. Although preprocessing methods such as normalization, resizing, segmentation, and data augmentation are widely used, many studies adopt general-purpose approaches that may not fully address the complex textural characteristics of honeycombing and GGO patterns. Inadequate preprocessing may result in the loss of fine-grained structural information and consequently limit the ability of models to learn discriminative features. This highlights the importance of investigating preprocessing strategies specifically tailored to lung CT imaging and the structural characteristics of interstitial lung disease patterns. Based on the reviewed evidence, the major research gaps can be summarized into four areas: (i) *limited emphasis on honeycombing and GGO classification*; (ii) *continued reliance on accuracy as the primary performance metric;* (iii) *limitations related to dataset size, class balance, diversity, and annotation quality*; and (iv) *insufficient optimization of preprocessing techniques specifically for lung CT imaging*. These gaps collectively affect the reliability, reproducibility, and generalizability of existing deep learning-based classification approaches.

Overall, the reviewed evidence demonstrates considerable potential for deep learning in automated lung disease classification using CT imaging, but important methodological and clinical challenges remain. Addressing these challenges requires more robust feature-learning approaches, comprehensive evaluation frameworks, domain-specific preprocessing strategies, and larger, diverse, and well-annotated CT datasets. In particular, greater attention to honeycombing and GGO differentiation is necessary to address an underexplored aspect of interstitial lung disease classification. Continued methodological development and clinical validation will be essential before automated lung CT classification systems can be reliably translated from experimental research settings into clinically useful computer-aided diagnostic applications. The research directions arising from these identified gaps are discussed in the following section.

## 5. Future Research Directions

The findings of this review highlight several areas that require further investigation to address the current limitations of deep learning-based lung disease classification using CT imaging. Future research should focus not only on improving classification performance but also on enhancing dataset quality, methodological consistency, model interpretability, computational efficiency, and clinical applicability. In particular, greater attention is needed to improve the differentiation of honeycombing and Ground-Glass Opacity (GGO) patterns, which remains challenging because of their overlapping visual characteristics. Based on the research gaps identified across the reviewed studies, several potential directions are discussed below.

### 5.1. Large-Scale and Multi-Institutional CT Datasets

Future research should prioritize the development of large-scale, multi-institutional CT datasets containing diverse patient populations and well-annotated lung disease patterns. The limited size and single-institution nature of many existing datasets may restrict model generalizability to unseen clinical environments. Standardized annotation protocols involving experienced radiologists could further improve the reliability of reference labels and support the development of more robust classification models. Multi-institutional datasets may also provide greater variation in patient characteristics, imaging protocols, and disease presentations, thereby supporting the development of models that are more robust across different clinical settings.

### 5.2. Standardized Preprocessing and Evaluation Frameworks

Greater standardization of preprocessing procedures, including image normalization, segmentation, resizing, augmentation, and region-of-interest selection, is needed to facilitate meaningful comparison between studies. Future studies should also adopt standardized evaluation frameworks incorporating multiple complementary metrics rather than relying primarily on accuracy. Reporting sensitivity, specificity, precision, F1-score, and AUC would provide a more comprehensive assessment of model performance, particularly when datasets contain imbalanced disease classes. Consistent reporting of preprocessing strategies, dataset partitioning, validation procedures, and evaluation metrics would further improve the reproducibility and comparability of future lung CT classification studies.

### 5.3. Improved Differentiation of Honeycombing and GGO

The differentiation of honeycombing and GGO remains an important research challenge because these patterns can exhibit overlapping visual characteristics in CT images. Future models should therefore investigate domain-specific preprocessing, advanced augmentation, and more effective feature-learning strategies to capture subtle structural differences between these patterns. Larger and more diverse datasets may also support the development of models that generalize more effectively across different disease presentations and imaging conditions. Further investigation into region-specific and multi-scale feature extraction may also help models distinguish subtle structural characteristics associated with honeycombing and GGO.

### 5.4. Explainable Artificial Intelligence

Explainable Artificial Intelligence (XAI) represents an important direction for improving the transparency and clinical acceptability of deep learning models. Future studies should investigate interpretable techniques that can identify and visualize the regions of CT images contributing to model predictions. XAI has been highlighted as an important approach for improving the transparency and interpretability of deep learning-based medical imaging systems [83]. Explainability is also important in healthcare applications because clinically meaningful interpretations can support understanding of AI model predictions and facilitate their integration into clinical workflows [84]. In addition, XAI has been investigated for cancer classification in medical imaging, demonstrating its potential to support more transparent and interpretable diagnostic systems [85]. Such approaches may help radiologists understand model reasoning, identify potentially incorrect predictions, and increase confidence in AI-assisted diagnostic systems. This is particularly relevant to honeycombing and GGO classification, where visual explanations could help determine whether the model is focusing on clinically meaningful structural regions.

### 5.5. Vision–Language Models and Multimodal Learning

Vision–language Models (VLMs) represent an emerging research direction for lung disease classification because they provide the potential to integrate visual information from medical images with textual clinical information. A vision–language pretrained transformer has been developed for respiratory healthcare using paired chest CT scans and radiology reports, demonstrating the potential of integrating CT imaging with textual clinical information for respiratory disease-related clinical tasks [86]. Vision–language foundation models have also been reviewed across medical imaging applications, including CT, with potential applications in disease classification, image interpretation, and report generation [87]. In addition, multimodal artificial intelligence has been investigated in medical diagnostics, highlighting the potential of combining heterogeneous clinical information to support more comprehensive medical decision-making [88]. These developments suggest that VLMs and multimodal learning could be explored for future honeycombing and GGO classification by integrating CT image features with radiological descriptions and relevant clinical information. Such multimodal approaches may provide more context-aware interpretation of subtle and overlapping interstitial lung patterns than image-only classification models. However, the application of VLMs specifically to honeycombing and GGO classification remains underexplored, highlighting the need for further investigation into domain-specific datasets, clinical validation, interpretability, and the reliability of multimodal outputs. A comprehensive review has further examined the evolution of vision–language models in medical image analysis, from early fusion-based approaches to general-purpose large models, providing a broader technical foundation that could inform the future application of VLMs to honeycombing and GGO classification [87,88].

### 5.6. Knowledge Distillation and Lightweight Models

Knowledge distillation and teacher–student learning frameworks represent promising directions for developing lightweight deep learning models while retaining diagnostic performance. Knowledge distillation has been applied to chest X-ray abnormality classification by transferring knowledge from larger teacher models to smaller student models, demonstrating its potential for multi-label thoracic disease classification [89]. Lightweight deep learning models using knowledge distillation have also been investigated for pulmonary disease detection in chest X-rays, demonstrating the potential to reduce computational requirements while maintaining classification performance [90]. In addition, knowledge distillation has been explored for chest X-ray diagnosis by transferring knowledge from a self-supervised teacher model to a compressed student model, supporting the development of computationally efficient medical imaging systems [91]. These findings suggest that knowledge distillation could be investigated for lung CT classification to reduce model complexity and facilitate deployment in real-time and resource-constrained clinical environments. Such approaches may be particularly useful when high-performing architecture requires substantial computational resources for training or inference. A comprehensive review of knowledge distillation and teacher-student learning in medical imaging has further outlined its pivotal role across diverse clinical imaging tasks and identified future research directions, offering additional support for its potential applicability to lung CT classification [92].

### 5.7. Federated Learning and Privacy-Preserving Collaboration

Federated learning provides another potential direction for addressing the limitations associated with institution-specific datasets. A collaborative federated learning framework has been investigated for lung and colon cancer classification, highlighting the potential of decentralized learning for medical image analysis while maintaining patient data privacy [93,94]. Privacy-preserving federated learning has also been investigated for multi-source electronic health records, enabling collaboration between multiple healthcare institutions while maintaining data locality and protecting sensitive patient information [95]. These findings suggest that federated learning could be explored for multi-institutional lung CT classification, particularly for honeycombing and GGO, where access to larger and more diverse datasets is important for improving model generalizability. Future research should investigate federated approaches that can address differences in imaging protocols, patient populations, annotations, and data distributions across healthcare institutions while maintaining patient privacy, ethical requirements, and regulatory compliance. A recently proposed personalized federated learning framework using perceptual hashing and a hypernetwork has demonstrated improved performance on heterogeneous medical image analysis tasks by jointly balancing personalization and generalization across clients, suggesting a promising direction for addressing data heterogeneity in future multi-institutional lung CT classification studies [96].

Collectively, these future research directions highlight the need to move beyond isolated image classification toward robust, interpretable, efficient, and clinically applicable AI systems. Integrating improved datasets, standardized methodologies, explainable models, multimodal learning, lightweight architectures, and privacy-preserving collaborative learning could contribute to the development of more reliable automated systems for honeycombing, GGO, and other interstitial lung disease patterns. Future investigations should therefore consider not only predictive performance but also model transparency, computational efficiency, data privacy, multimodal clinical context, and generalizability across healthcare institutions. Such developments could support the eventual translation of automated lung CT classification systems from experimental research settings into clinically useful decision-support applications.

## Figures and Tables

**Figure 1:**
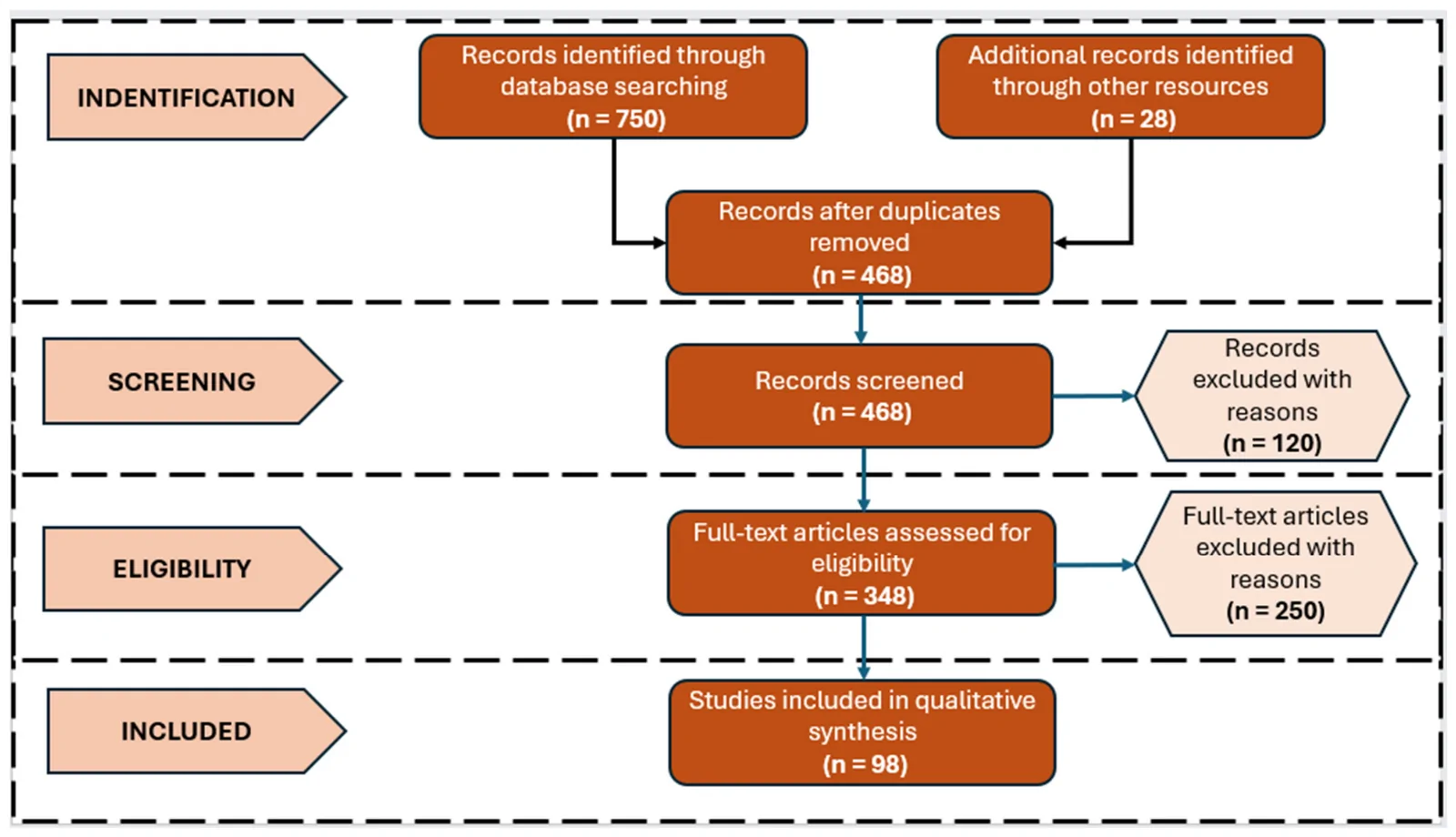
PRISMA Flowchart for Systematic Review.

**Table 1:** Sources of Scientific Literature for Study Selection (2011–2026).

Online Database	Simplified Description
IEEE Xplore Digital Library (IEEE)	A leading database for engineering and computing research, widely used for studies on deep learning, medical imaging, and algorithm development.
ScienceDirect	A large collection of peer-reviewed journals covering engineering, life sciences, health sciences, and multidisciplinary research articles.
SpringerLink	A comprehensive academic platform providing access to journals, books, and conference proceedings across science, technology, and medicine.
PubMed	A biomedical database maintained by the U.S. National Library of Medicine, focusing on life sciences and clinical research literature.
Scopus	One of the largest citation databases is indexing journals, conference papers, and books across multiple scientific disciplines.
Web of Science	A multidisciplinary citation database used for tracking high-impact journals and research trends across science and engineering fields.
ACM Digital Library	A specialized repository for computer science and IT research, including publications in artificial intelligence and software engineering.
MDPI	An open-access publishing platform that provides peer-reviewed journals covering multiple disciplines, including medical imaging, artificial intelligence, and healthcare.
Google Scholar	A freely accessible search engine that indexes scholarly literature across multiple disciplines and publication formats.

**Table 2:** Summary of Medical Imaging Modalities for Lung Disease Diagnosis.

Imaging Modality	Simplified Description
X-ray	Commonly used to detect lung abnormalities such as pneumonia; simple and cost-effective.
CT	Provides high-resolution, detailed cross-sectional images useful for identifying lung diseases.
MRI	Produces detailed soft-tissue images, but less common due to quality and data limitations.
PET	Used mainly for functional and metabolic imaging; limited studies on lung classification.

**Table 3:** Summary of Publicly Available and Exclusive Datasets for Lung Disease Analysis.

Ref.	Year	Category	Classes	Data Size	Annotation Method	Database Collection Strategy	Class Distribution	Image Modalities
[30]	2016	Classification	Healthy, Honeycombing, GGO, Reticular, Emphysema, Micronodules	14,696	Radiologist annotated	Clinical dataset	Not reported	CT
[31]	2017	Classification	Benign, Malignant	14,520	Radiologist annotated	Public database	Imbalanced	CT
[32]	2022	Classification	Benign, Malignant	8474	Radiologist annotated	Public database	Imbalanced	CT
[33]	2019	Segmentation/localization	Honeycombing	Not reported	Not reported	Clinical dataset	Not reported	CT
[34]	2021	Classification	Healthy/Pneumonia	6000	Pre-labelled	Public Database	Imbalanced	X-Ray
[35]	2019	Segmentation and Classification	Emphysema, honeycombing, pulmonary consolidation, ground glass opacity	1218	Radiologist-verified	Clinical dataset	Imbalanced	CT

**Table 4:** Image Preprocessing Methods for Lung Disease Classification: Representative Studies.

Preprocessing Method	Definition/Methodology	Advantages	Recent Studies
Image Augmentation	Geometric and intensity-based transformations such as rotation, flipping, scaling, cropping, and noise injection to generate additional training samples.	Increases dataset diversity, reduces overfitting, and improves model generalization, particularly for limited or imbalanced datasets.	[31,39,40,41,42]
Image Normalization	Standardizes image intensity values using statistical scaling, intensity correction, or normalization methods to reduce variation between images and improve consistency across datasets.	Improves training stability, reduces intensity variability, and enhances model generalization, particularly for multi-site or heterogeneous medical imaging datasets.	[36,37,38]
Image Scaling	Resizing images using interpolation methods to obtain consistent input dimensions while preserving relevant anatomical structures.	Ensures uniform model input size, improves computational efficiency, and facilitates stable training across different imaging datasets.	[37,39,45,46,48,49]
Regions of Interest (ROI)	Selects or isolates disease-relevant anatomical regions using localization, patch extraction, thresholding, or segmentation-based region selection techniques.	Reduces irrelevant background information, focuses model learning on clinically important regions, and improves feature representation.	[16,35,43,44]
CLAHE Enhancement	Enhances local contrast using Contrast Limited Adaptive Histogram Equalization to improve visibility of subtle anatomical and pathological structures.	Improves local contrast, highlights subtle abnormalities, and enhances feature representation for deep learning models.	[16,17]
Segmentation	Extracts lung regions or pathological structures using classical image processing or deep learning-based segmentation methods.	Removes irrelevant anatomical regions, improves focus on disease-related structures, and enhances feature learning.	[16,19,45,46,47]
Denoising	Applies noise reduction techniques to suppress image artifacts while preserving important diagnostic structures, particularly in low-dose CT imaging.	Improves image quality, reduces acquisition noise, and enhances the reliability of extracted features.	[18]

**Table 5:** Evaluation Metrics Reported in Representative Lung Disease Classification Studies.

Study Reference	Accuracy (Ac)	Precision (Pr)	Sensitivity (Sn)	Specificity (Sp)	AUC-ROC
[50]	√	√	√	√	√
[51]	√	√	√	√	√
[52]	√	√	√	√	√
[53]	√	-	√	√	√
[48]	√	-	√	√	-
[30]	√	-	√	√	√
[54]	√	√	√	-	√
[55]	√	-	√	-	-
[56]	√	√	√	√	√
[57]	√	√	√	√	√
[58]	√	√	√	√	√
[59]	√	√	√	-	√
[60]	√	√	√	√	√
[61]	√	-	√	-	-
[62]	√	√	-	-	√
[63]	√	√	√	√	-
[3]	√	-	-	-	-
[33]	√	-	-	-	-
[34]	√	√	√	-	-

Note: √ indicates that the corresponding performance metric was reported in the referenced study.

**Table 6:** Classifier-Based Approaches for Lung Disease Classification: A Summary of Representative Studies from the Included Literature.

Study Reference	ML Model	Lung Diseases	Performance Metric
[2]	csSVM, SVM	Diffuse Interstitial Lung Diseases (DILD)	Ac: 89.88%
[64]	SVM/Bayesian Classifier	Diffuse Interstitial Lung Disease (DILD)	Ac: 92%(SVM)/77%(Bayesian)
[65]	SVM, RF, KNN, DT	Lung Cancer	Ac: 99.32%, Pr: 99%, Rc: 99%, F1: 99%
[66]	SVM + Feed-Forward Backpropagation Neural Network (FFBPNN)	Lung Cancer	Ac: 98.08%, Pr: 98.17%, Rc: 96.50, F1: 97%
[67]	KNN	Tuberculosis (TB)	Ac: 71.81%
[68]	KNN	COVID-19	Ac: 96%
[69]	KNN	Lung Cancer, GGO, Pulmonary Fibrosis, Healthy	Ac: 94%
[56]	Multiple Instance Learning (MIL), Active Learning (AL)	Tuberculosis (TB)	AUC: 0.71

**Table 7:** Deep Learning Architectures and Reported Performance Metrics for Lung Disease Classification: Representative Studies from the Included Literature.

Study Reference	DL Model	Lung Diseases	Performance Metrics
[33]	CNN	Normal, GGO, Honeycombing	Ac: Healthy 87.8%, GGO 73.3%, HC 69.1%
[34]	ResNet50/DenseNet201/InceptionV3/GoogleNet/AlexNet	Normal and Pneumonia	Ac: 97.22%, Sn: 95.78%, Sp: 97.69%, F1: 94.51% (Best ResNet50 results)
[72]	ResNet50/ResNet101/ResNet152	Normal, Pneumonia, GGO and COVID-19	F1:ResNet50 (91%), ResNet101 (93%), ResNet152 (94%)
[73]	Multi-task CNN (Encoder–Decoder)	COVID-19, Normal, Pneumonia	AC: 94.67%, Dice: 88%, AUC: >97%
[74]	CNN	COVID-19, Pneumonia, Normal	Binary: Ac 98.08%, Sn 95.13%, Sp 95.30%, Precision 98.03%, F1 96.51%; Multi-class: Ac 87.02%, Sn 85.35%, Sp 92.18%, Precision 89.96%, F1 87.37%
[57]	VGG16/ResNet-50/InceptionV3 + U-Net segmentation	Pneumonia, Tuberculosis, Normal	Ac: 82%; Healthy—AUC: 90%, F1: 76%, Pr: 75%, Sn: 77%; Pneumonia—AUC: 99%, F1: 93%, Pr: 90%, Sn: 95%; Tuberculosis—AUC: 93%, F1: 78%, Pr: 81%, Sn: 75% (Best InceptionV3 results)
[75]	DenseNet201	Normal, Pneumothorax, Atelectasis	Ac: 95.34%, Pr: 97%, Rc: 96%, F1: 96%
[76]	VGG16 + PCA	Multiple Thoracic Diseases	Ac: 96%
[77]	VGG16/CNN/ResNet50/InceptionV3/MobileNetV2/	Normal, Benign, Malignant	A:98.18% (Best VGG16 results)
[78]	VGG16 + CNN	Lung Cancer	Ac:92.53%
[79]	ResNet-50	Normal, Benign, Malignant	Ac: 98.77%
[17]	Swim Transformer/XGBoost	Benign, Malignant	Ac: 95.8%
[49]	EfficientNet-B7/VGG-16	Lung Cancer	Ac: 98.73%
[80]	VGG16	Histopathological Lung Disease	Ac: 92%

## Data Availability

The data that supports the findings of this study are available on request.
